# Rare pancreatic ductal adenocarcinoma variants and other malignant epithelial tumors: a comprehensive clinical and radiologic review

**DOI:** 10.1007/s11604-025-01777-7

**Published:** 2025-04-11

**Authors:** Yukihisa Takayama, Ryo Murayama, Shinji Tanaka, Keisuke Sato, Kazuki Goto, Gaku Honda, Kengo Yoshimitsu

**Affiliations:** Department of Radiology, Faculty of Medicine, Fukuoka University, 7-45-1 Nanakuma, Jonan-ku, Fukuoka City, Fukuoka 814-0180 Japan

**Keywords:** Pancreatic ductal adenocarcinoma, Rare histologic subtype, CT, MRI, ^1^⁸F-FDG-PET, Radiological features

## Abstract

Over 95% of pancreatic carcinomas are classified as conventional pancreatic ductal adenocarcinoma (cPDAC), while less than 5% consist of rare histological subtypes. Some of these rare histological subtypes, such as colloid carcinoma, medullary carcinoma, and undifferentiated carcinoma with osteoclast-like giant cells, are associated with a relatively better prognosis compared to cPDAC, whereas others, including signet ring cell carcinoma/poorly cohesive carcinoma, adenosquamous carcinoma, large cell carcinoma with rhabdoid phenotype, and undifferentiated carcinoma, have a worse prognosis. Other malignant pancreatic epithelial tumors (MPET) include acinar cell carcinoma, pancreatoblastoma, and solid-pseudopapillary neoplasm that should also be differentiate from PDACs. Accurate differentiation among PDAC subtypes and other MPETs is essential for precise survival predictions and effective therapeutic planning. However, cPDAC, rare histological subtypes of PDAC and MPETs often exhibit similar imaging findings, making it challenging to establish a diagnosis based solely on imaging. Thus, needle biopsy or surgical resection is generally required for the final diagnosis. We herein present a review article based on case studies and literature reviews of rare histological subtypes of PDAC and other MPET, with particular focus on their imaging characteristics, referencing the 5th edition of the World Health Organization classification.

## Introduction

### Epidemiology and subtypes of pancreatic ductal adenocarcinoma (PDAC)

According to the Vital Statistics of Japan reported by the Ministry of Health, Labour and Welfare, PDAC ranked as the fourth leading cause of cancer-related deaths in men and the third in women in 2022 [1]. PDAC encompasses several histologic subtypes, including conventional PDAC (cPDAC), colloid carcinoma (CC), signet ring cell carcinoma (SRCC)/poorly cohesive carcinomas (PCC), adenosquamous carcinoma (ASqC), medullary carcinoma (MC), hepatoid carcinoma (HC), large cell carcinoma with rhabdoid phenotype (LCC-RP), undifferentiated carcinoma (UC), and UC with osteoclast-like giant cells (UCOGC) [2]. Epidemiological data from the Surveillance, Epidemiology, and End Results program in the United States indicate that among 159,548 patients with PDAC, cPDAC constitutes 95.9%, while other subtypes are distributed as follows: CC (2.6%), ASqC (0.8%), SRCC (0.5%), UC (0.1%), UCOGC (0.1%), HC (0.01%), and MCP (0.006%) [3]. Other malignant pancreatic epithelial tumors (MPETs) include acinar cell carcinoma (ACC), pancreatoblastoma (PB), and solid-pseudopapillary neoplasm (SPN) [2].

## Imaging protocols for pancreatic tumor

### Computed tomography (CT)

CT is performed with thin slices (1–3 mm), with even thinner slices (0.5–1.25 mm) preferred for three-dimensional (3D) reconstruction [4]. A high-concentration contrast agent (600 mg iodine/kg) is administered over approximately 30 s, with imaging timing determined by bolus tracking. Following a non-contrast-enhanced CT (non-CECT), a dynamic contrast-enhanced CT (DCE-CT) is performed in four phases using a high-concentration contrast injection: early arterial phase (defined as a 100 Hounsfield unit increase in the descending aorta), pancreatic phase (45 s post-injection), portal venous phase (around 60 s), and delayed phase (240 s) [4].

### Magnetic resonance imaging (MRI)

Standard MRI sequences include T1-weighted imaging (T1WI), fat-suppressed T1WI, T2-weighted imaging (T2WI) or fat-suppressed T2WI, diffusion-weighted imaging (DWI), apparent diffusion coefficient (ADC) maps, and two-dimensional or 3D magnetic resonance cholangiopancreatography (MRCP) [4]. Chemical-shift imaging is employed to evaluate focal fat content [4]. For dynamic imaging using extracellular or hepatocyte-specific contrast agents, 3D fat-suppressed T1WI is performed [4].

Both CT and MRI play essential roles in assessing tumor characteristics, tumor extent, lymph-node involvement, and distant metastases.

### ^18^F-fluoro-2-deoxy-D-glucose positron emission tomography (^18^F-FDG-PET)

^18^F-FDG-PET or ^18^F-FDG-PET with CT (^18^F-FDG-PET/CT) is not routinely indicated for pancreatic tumor evaluation but is recommended in cases where distant metastases are suspected on CT.

## Conventional PDAC

cPDAC, which accounts for 95.9% of all pancreatic malignancies, is a malignant epithelial neoplasm characterized by pancreatic ductal differentiation and mucin production [3, 5]. The median age at diagnosis is 68 years, with 48.2% of patients being female [3]. The median survival is approximately 8 months, and the 5-year survival rate is only 5.1% [3]. Abdominal pain is the most commonly reported symptom, even in tumors smaller than 2 cm [5]. Other symptoms—including back pain, weight loss, loss of appetite, and jaundice—are more frequently observed in advanced stages. Elevated tumor markers, such as carbohydrate antigen 19–9, carcinoembryonic antigen (CEA), sialylated pancreatic antigen-1, and Duke pancreatic monoclonal antigen type 2, have been observed in patients with cPDAC. While the sensitivity of these tumor markers is high, their specificity remains relatively low, with a false-positive rate of 20–30% [6].

**Fig. 1:**
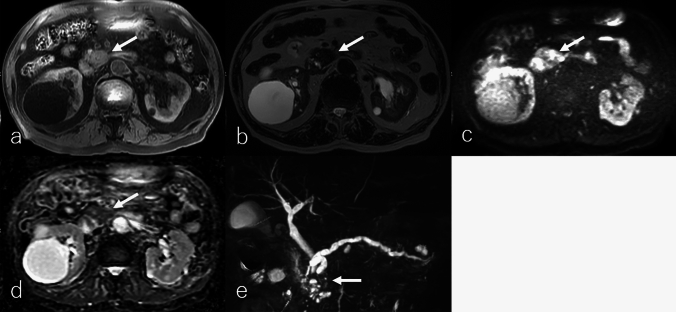
An 80-year-old female patient with conventional pancreatic ductal adenocarcinoma (cPDAC). **a** Fat-suppressed T1-weighted imaging (FS-T1WI), **b** T2-weighted imaging (T2WI), **c** diffusion-weighted imaging (DWI) with *b*-value of 800 s/mm^2^, **d** apparent diffusion coefficient (ADC) map, and magnetic resonance cholangiopancreatography (MRCP). The tumor is located in the pancreatic uncinate process (arrow), measuring 3.0 cm in diameter, and exhibits infiltrative growth with partially ill-defined borders. The tumor exhibits heterogeneous isointensity on FS-T1WI and T2WI, heterogeneous hyperintensity on DWI, and slightly lower ADC values (1.02 × 10^−3^ mm^2^/s) relative to the pancreas parenchyma. MRCP shows that the tumor-induced obstruction of the pancreatic and/or bile ducts results in upstream ductal dilation and retention cyst formation

Radiologically, cPDAC typically appears as an ill-defined, poorly enhancing mass, often associated with ductal obstruction, obstructive pancreatitis, and segmental pancreatic atrophy [5]. On MRI, it demonstrates iso- to hypointensity on T1WI, variable signal intensity on T2WI, homogeneous hyperintensity on DWI, and a lower ADC relative to the pancreatic parenchyma (Fig. 1a–d). However, its signal characteristics may vary depending on the presence of necrosis, mucin content, or fibrotic stromal tissue within the tumor. The tumor-induced obstruction of the pancreatic and/or bile ducts results in upstream ductal dilation and retention cyst formation (Fig. 1e).

**Fig. 2:**
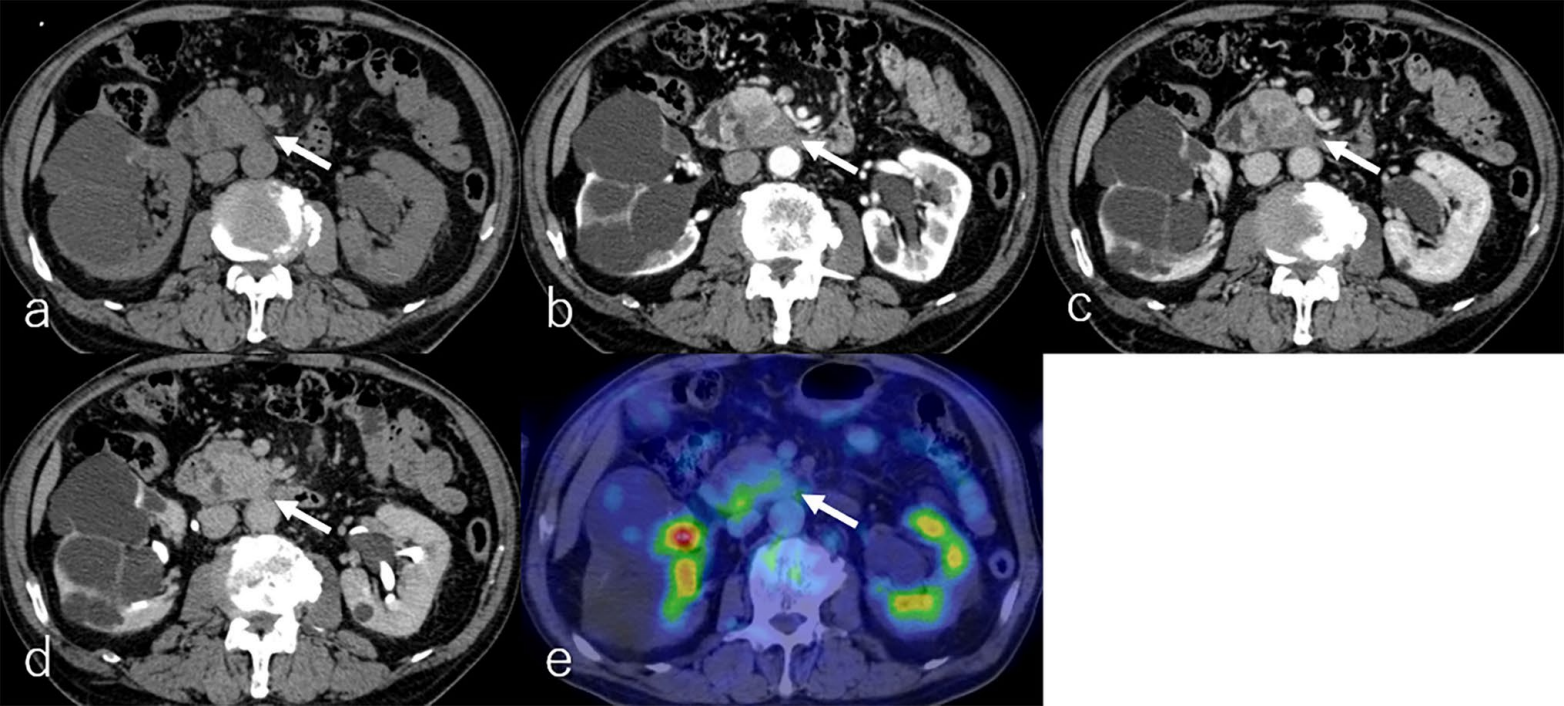
An 80-year-old female patient with conventional pancreatic ductal adenocarcinoma (cPDAC), the same patient as in Fig. 1. **a** Non-contrast-enhanced computed tomography (non-CECT) and dynamic contrast-enhanced CT in the **b** pancreatic phase, **c** portal phase, and **d** delayed phase, and **e** ^18^F-fluoro-2-deoxy-D-glucose positron emission tomography with CT (^18^F-FDG-PET/CT). The tumor is located in the pancreatic uncinate process (arrow), measuring 3.0 cm in diameter, and exhibits infiltrative growth with partially ill-defined borders. It appears isoattenuation on non-CECT and demonstrates gradual delayed enhancement during the portal and delayed phases, suggesting intratumoral fibrotic stroma, a characteristic finding of cPDAC. On ^18^F-FDG-PET/CT, the tumor shows mildly increased ^18^F-FDG uptake, with a maximum standardized uptake value of 2.9

^1^⁸F-FDG-PET or PET/CT typically demonstrates increased ^18^F-FDG uptake in cPDAC [7]. However, tumors with abundant fibrotic stroma or small lesions may exhibit low ^1^⁸F-FDG uptake (Fig. 2e). Additionally, patients with cPDAC frequently have diabetes, which can lower ^1^⁸F-FDG uptake in the tumor and contribute to false-negative results [8].

On non-CECT, cPDAC typically appears isoattenuating relative to the pancreatic parenchyma (Fig. 2a). In DCE studies, it exhibits poor enhancement during the pancreatic phase, while progressive enhancement in the portal and delayed phases reflects the presence of abundant fibrous stroma (Fig. 2b–d). Notably, approximately 10% of PDACs remain isoattenuating to the pancreatic parenchyma in DCE studies due to minimal desmoplastic stroma, making detection challenging [5].

## Challenges in diagnosis

Pathological examination remains essential for definitive tumor diagnosis, whereas imaging plays a critical role in evaluating pancreatic malignancies by characterizing tumors, distinguishing benign from malignant lesions, assessing invasion and resectability, guiding treatment, and predicting prognosis [9, 10]. Accurately differentiating histologic subtypes of PDACs and MPETs is crucial for precise prognostic assessment and the development of effective therapeutic strategies. However, rare histologic subtypes of PDAC and other MPETs often closely resemble cPDAC, complicating differential diagnosis. As a result, imaging alone has inherent limitations, and pathological confirmation is necessary in cases of diagnostic uncertainty. While identifying imaging features specific to rare subtypes could enhance diagnostic accuracy, histopathological confirmation via biopsy or surgical resection is often required for definitive classification. This review synthesizes case studies and existing literature on the clinical and radiological features of PDAC subtypes and other MPETs, incorporating references from the 5th edition of the World Health Organization (WHO) classification (Tables 1, 2, 3) [2]. Due to manuscript length constraints, descriptions of neuroendocrine neoplasms (NEN) have been omitted.

**Table 1:** Malignant epithelial tumors of the pancreas in the 5th edition of the WHO classification

Duct adenocarcinoma NOS
Colloid carcinoma
Poorly cohesive carcinoma
Signet ring cell carcinoma
Medullary carcinoma
Adenosquamous carcinoma
Hepatoid carcinoma
Large cell carcinoma with rhabdoid phenotype
Carcinoma, undifferentiated NOS
Undifferentiated carcinoma with osteoclast-like giant cells
Acinar cell carcinoma
Acinar cell cystadenocarcinoma
Mixed acinar-neuroendocrine carcinoma
Mixed acinar-endocrine-ductal carcinoma
Mixed acinar-ductal carcinoma
Pancreatoblastoma
Solid-pseudopapillary neoplasm of the pancreas
Solid-pseudopapillary neoplasm with high-grade dysplasia

*WHO* World Health Organization, *NOS* Not otherwise specified

**Table 2:** Clinical features of subtypes of PDAC and MPETs

Subtype	Frequency	Median age (years)	Sex Predominance	Predilection site	Prognosis vs. cPDAC	Other features
CC	2.6% of PDACs	68	Slight female	Head	Better	Trousseau syndrome may be induced by surgery or biopsy
SRCC/PCC	0.5% of PDACs	68	Male	Head	Worse	Gastric mucosa heterotopia/metaplasia may contribute
MC	0.006% of PDACs	65	Female	Mostly head	Highest survival among PDAC subtypes, but limited by low case numbers	Indolent course
ASqC	0.8% of PDACs	68	Male	Mostly head	Worse	Malignancy-related hypercalcemia was reported
HC	~ 0.01% of PDACs	65	Male	Body & tail	Better, but prediction is limited by low cases	Early hematogenous liver metastasis, lymph-node involvement, elevated AFP/des-gamma-carboxyprothrombin
LCC-RP	~ 0.003% of PDACs	61	Female	Mostly body & tail	Worst among PDAC subtypes, but limited by low cases	Rapidly fatal
UC	~ 0.1% of PDACs	67	Male	Head	Variable among subtypes	–
UCOGC	~ 0.1% of PDACs	66	Slight female	Body & tail	Better	–
ACC	1–2% of adult pancreatic tumors; ~ 15% pediatric	60	Male	Head	The prognosis is controversial (reports of both better and worse)	~ 16% present with disseminated fat necrosis
PB	Rare, < 200 cases reported	4 (mostly < 10 years)	Slight male	No preference	Worse in adults than in pediatrics	Elevated AFP
SPN	3% of all pancreatic tumors; 6% of exocrine tumors	26 (mostly 20 s-30 s)	90% Female	Head	Generally good	~ 15% show aggressive features (organ invasion/metastasis)

*PDAC* pancreatic ductal adenocarcinoma; *MPET* malignant pancreatic epithelial tumor; *cPDAC* conventional pancreatic ductal adenocarcinoma; *CC* colloid carcinoma; *SRCC* signet ring cell carcinoma; *PCC* poorly cohesive carcinomas; *MC* medullary carcinoma; *ASqC* adenosquamous carcinoma; *HC* hepatoid carcinoma; *LCC-RP* large cell carcinoma with rhabdoid phenotype; *UC* undifferentiated carcinoma; *UCOGC* undifferentiated carcinoma with osteoclast-like giant cell; *ACC* acinar cell carcinoma; *PB* pancreatoblastoma; *SPN* solid-pseudopapillary neoplasm; *AFP* alfa fetoprotein

**Table 3:** Radiological features of subtypes of PDAC and MPETs

Subtype	Morphology	T1WI/FS-T1WI*	T2WI/FS-T2WI*	DWI*	ADC*	Non-CECT*	DCE study*	^18^F-FDG-PET*	Key features for differential diagnosis
CC	Lobulated/indistinct margins	Hypointense	Hyperintense, "salt and pepper"	Hyperintense	High	Hypoattenuation	Sponge-like progressive delayed enhancement	Increased uptake	Excessive mucin, distinct imaging from cPDAC
SRCC/PCC	Focal/diffuse infiltration	Heterogeneous hypointense	Heterogeneous hyperintense	Heterogeneous hypointense	Low	Hypoattenuation	Gradual enhancement	N/A	Lacks specific features
MC	Well-defined margin	Heterogeneous hypointense	Heterogeneous hyperintense	N/A	N/A	Hypoattenuation	N/A	N/A	Challenging to differentiate from poorly differentiated cPDAC
ASqC	Well/ill-defined, large, round	Central necrosis: hetero hypointense, peripheral: hetero hypointense	Central necrosis: hetero hyperintense, peripheral: hetero hyperintense	Central necrosis: hyperintense, peripheral: hyperintense	Central: high, peripheral: low	Central: hypoattenuation, peripheral: isointense	Central: no enhancement, peripheral: progressive enhancement	Central: no uptake, peripheral: increased uptake	Extensive necrosis, ring enhancement, tumor thrombus
HC	Well-defined, large, exophytic	N/A	Heterogeneous iso-/hyperintense	Heterogeneous iso-/hyperintense	N/A	Hypoattenuation	Arterial enhancement, portal/delayed washout	Increased uptake	Rim encapsulation
UC	Expansive growth	Mixed iso-/hypointense	Heterogeneous hyperintense	N/A	N/A	N/A	Peripheral enhancement (portal venous phase)	Increased uptake	Large tumor, direct invasion
UCOGC	Well-defined, smooth margin, large	Heterogeneous hypointense	Heterogeneous hypointense	Heterogeneous hypointense	Low	Isoattenuation	Weak enhancement in all phases, prolonged enhancement	Increased uptake	Necrosis, hemosiderin deposition, intraductal/ intravenous growth
ACC	Well-defined, large, solid, minimal cystic changes	Heterogeneous hypointense	Heterogeneous iso-/hyperintense	Heterogeneous hyperintense	Low	Hypoattenuation (varies by size/necrosis)	Weak enhancement, early arterial enhancement with washout	Increased uptake	Necrosis, intraductal/ intravenous growth, calcifications (7–50%)
PB	Well-defined, large, lobulated	Heterogeneous hypo-/isointense	Heterogeneous hyperintense	Solid: hyperintense	Solid: low	Hypo-/isoattenuation	Large: heterogeneous, small: homogeneous progressive enhancement	Increased uptake	Patient's age, rim/clustered calcifications
SPN	Large, well-defined (mean 9–10 cm, smaller cases < 5 cm)	Heterogeneous hypo (41%) or hyper (12%)	Heterogeneous hyper (94%)	Heterogeneous hyper, hemorrhagic fluid–fluid level	Solid: low	Hypoattenuation ± peripheral calcification	Heterogeneous peripheral or homogeneous enhancement (arterial phase), progressive but incomplete enhancement	Uptake varies by cellularity	Hemorrhage (50%), "eggshell" calcification (33%)

*PDAC* pancreatic ductal adenocarcinoma; *MPET* malignant pancreatic epithelial tumor; *cPDAC* conventional PDAC; *CC* colloid carcinoma; *SRCC* signet ring cell carcinoma; *PCC* poorly cohesive carcinoma; *MC* medullary carcinoma; *ASqC* adenosquamous carcinoma; *HC* hepatoid carcinoma; *UC* undifferentiated carcinoma; *UCOGC* undifferentiated carcinoma with osteoclast-like giant cells; *ACC* acinar cell carcinoma; *PB* pancreatoblastoma; *SPN* solid-pseudopapillary neoplasm; *T1WI* T1-weighted imaging; *FS-T1WI* fat-suppressed T1WI; *T2WI* T2-weighted imaging; *FS-T2WI* fat-suppressed T2WI; *DWI* diffusion-weighted imaging; *ADC* apparent diffusion coefficient; *CT* computed tomography; *Non-CECT* noncontrast-enhanced CT; *DCE* dynamic contrast-enhanced; ^18^*F-FDG-PET* ^18^F-fluoro-2-deoxy-D-glucose PET

*Signal intensity, attenuation, and ^18^F-FDG uptake of tumors were compared to those of the pancreatic parenchyma

## Rare pancreatic ductal adenocarcinoma variants

### Colloid carcinoma

CC is characterized by at least 80% of the neoplastic epithelium suspended in extracellular mucin pools [2]. This subtype has been referred to by various synonyms, including mucinous noncystic carcinoma, gelatinous adenocarcinoma, and mucoid carcinoma [11]. It accounts for 2.6% of PDACs and is diagnosed at a median age of 68 years, with 50.2% of patients being female [3]. A report indicates that CC follows an indolent course, with a reported 5-year survival rate of 57–72% following surgical resection [12]. However, statistic primarily reflects postoperative survival rates for invasive intraductal papillary mucinous neoplasm (IPMN) and IPMN-associated invasive adenocarcinoma, rather than CC itself. Regarding tumors pathologically diagnosed as CC, studies by Adsay NV et al. and Seidel G et al. have reported significantly lower 5-year survival rates of 28% and 29%, respectively [13, 14]. Furthermore, a recent study reported that the median survival rate for patients with CC is 9 months, with a 5-year survival rate of 10.6%, demonstrating a relatively better prognosis than cPDAC [3]. CC often arises in association with intestinal-type IPMN, highlighting a potential precursor lesion [11]. Notably, CC can lead to migratory thromboembolism, termed “Trousseau syndrome,” particularly following surgical or needle biopsies, which may occasionally result in fatal outcomes [5].

**Fig. 3:**
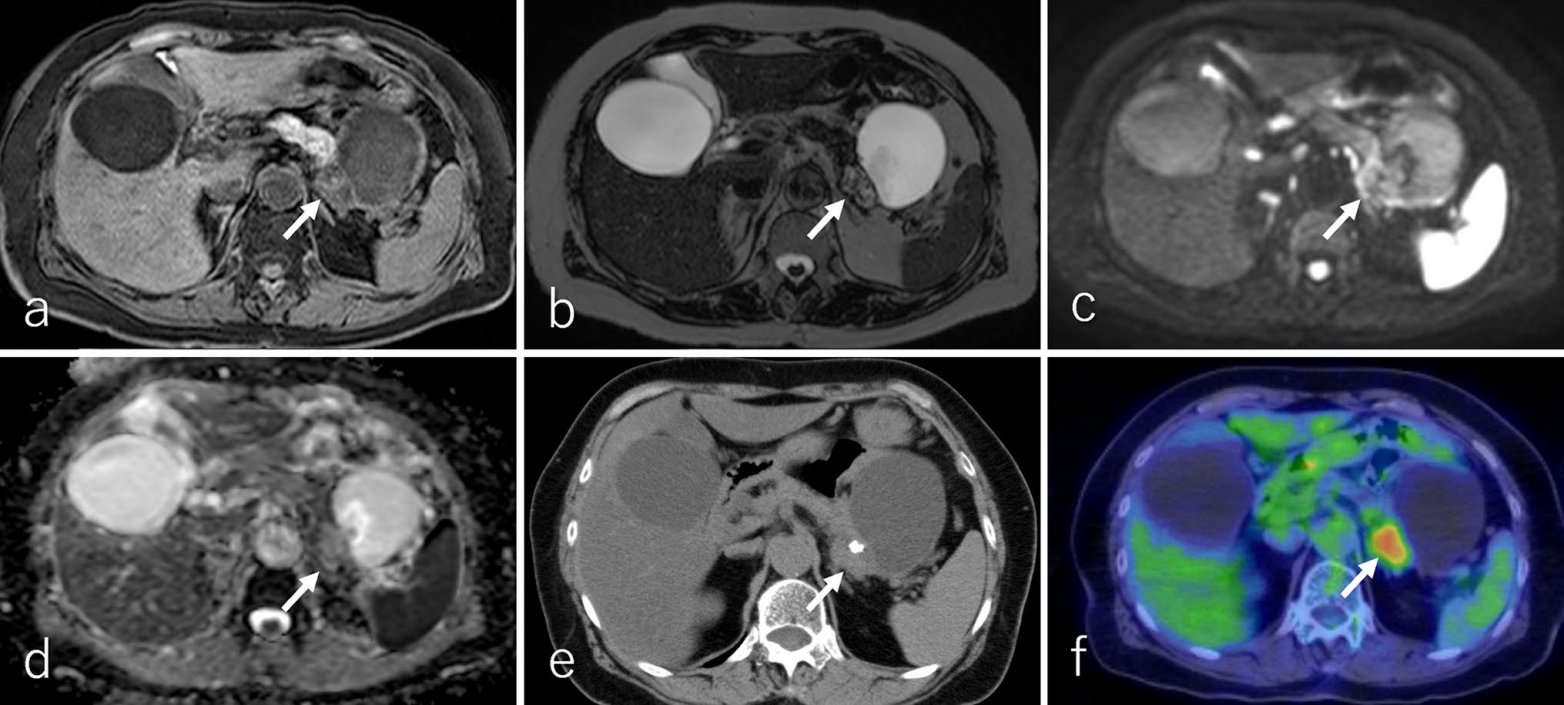
A 79-year-old female patient with colloid carcinoma (CC). **a** Fat-suppressed T1-weighted imaging (FS-T1WI), **b** T2-weighted imaging (T2WI), **c** diffusion-weighted imaging (DWI) with b value of 800 s/mm^2^, **d** apparent diffusion coefficient (ADC) map, **e** non-contrast-enhanced computed tomography (non-CECT) and **f** ^18^F-fluoro-2-deoxy-D-glucose positron emission tomography with CT (^18^F-FDG-PET/CT). The tumor is located in the pancreatic tail (arrow), measuring 3.0 cm in diameter, and demonstrates an ill-defined hypointensity on FS-T1WI, "mesh-like" mild hyperintensity on T2WI and DWI, higher ADC values (1.74 × 10^−3^ mm^2^/s), and hypoattenuation on non-CECT relative to the pancreatic parenchyma. On ^18^F-FDG PET/CT, the tumor exhibits increased ^18^F-FDG uptake, with a maximum standardized uptake value max of 4.30. A well-defined adjacent cyst, identified as a retention cyst, coexists with the CC but is not a pathologically neoplastic lesion

CC typically manifests with lobulated or indistinct margins [5]. It is more frequently located in the pancreatic head than the body or tail [3]. Due to excessive mucin production, CC may exhibit a cystic appearance despite not being a true cystic tumor, often leading to misinterpretation as primarily cystic lesions such as IPMN or mucinous cystic neoplasm (MCN) [5, 15]. The abundant mucin within CC contributes to its characteristic imaging findings, including hypointensity on T1WI, hyperintensity on T2WI and DWI, a higher ADC (Fig. 3a–d), and hypoattenuation on non-CECT relative to the pancreatic parenchyma (Fig. 3e) [15]. A distinctive “salt and pepper” appearance on T2WI, characterized by hyperintense masses with tiny dot-like hypointense foci, is a notable MRI feature of CC [15]. In DCE studies, CC demonstrates peripheral and internal sponge-like or mesh-like progressive delayed contrast enhancement [15]. Although ^18^F-FDG-PET or PET/CT reveals increased ^18^F-FDG uptake in CC, this finding is nonspecific and does not provide additional diagnostic value beyond MRI and CT (Fig. 3f) [16].

CC and cPDAC exhibit distinct imaging characteristics, particularly in T2WI signal intensities, which can aid differentiation. IPMN is the most critical entity to distinguish from CC among pancreatic neoplasms. IPMNs typically demonstrate communication between the mass and the pancreatic duct, downstream dilatation of the pancreatic duct, and intraductal papillary components—features absent in CC [5]. MRCP is a valuable tool for evaluating these features and differentiating CC from cPDAC and IPMN [5, 15]. MCN, another important differential diagnosis, is characterized by large, unilocular, or septated macrocystic lesions with intracystic enhancing soft tissue and a thickened capsular rim, contrasting with the indistinct margins and gradual internal enhancement typical of CC [5].

### Signet ring cell carcinoma/poorly cohesive carcinoma

SRCC/PCC is a neoplasm with a predominantly dyshesive growth pattern with single-cell or cord-like stromal infiltration [17]. While SRCC/PCC commonly arises in the stomach—especially among Japanese patients—it has also been documented in the colon, rectum, gallbladder, ampulla of Vater, and pancreas [2]. Although some studies suggest SRCC as a distinct subtype of PCC, the WHO classification treats them as identical identities [2, 18, 19]. To date, pancreatic PCC has not been reported; therefore, this review discusses SRCC and PCC collectively as SRCC [2].

SRCC consists of at least 80% poorly cohesive cells containing intracellular mucin vacuoles and displaced nuclei [2, 20]. It accounts for 0.5% of PDACs with a median diagnostic age of 68, and 40.3% of cases occur in females [3]. The prognosis for SRCC is poor, with a median survival of 5 months and a 5-year survival rate of 3.4%, significantly worse than that of cPDAC [3]. SRCC metastasizes to atypical sites, such as bone and leptomeninges, and frequently involves the lungs, which is rare in cPDAC [18, 21]. Widespread metastases are the primary contributor to its dismal prognosis [18]. While the etiology remains unclear, heterotopic gastric mucosa near the ampulla of Vater is hypothesized to play a role [5].

Case reports on imaging findings for pancreatic SRCC are limited. One report described isolated dilatation of the common bile duct without an associated mass, while another noted diffuse pancreatic infiltration [21, 22]. SRCC typically occurs in the pancreatic head or tail, often near the duodenal papilla [3, 5]. On imaging, SRCC exhibits heterogeneous hyperintensity on fat-suppressed T2WI and DWI, a lower ADC relative to the pancreatic parenchyma, and poor enhancement on CECT [5]. DCE studies demonstrate gradually increasing enhancement [23, 24]. Although no pancreatic SRCC cases have been reported using ^18^F-FDG-PET or PET/CT, data from gastric SRCC suggest low ^1^⁸F-FDG uptake, likely due to its morphological characteristics [25, 26].

The imaging findings of SRCC lack specificity, complicating differentiation from cPDAC [5]. Metastatic extrapancreatic SRCC should be excluded prior to diagnosing pancreatic SRCC [2]. Additionally, CC should be considered in the differential diagnosis, as it may contain individual signet ring cells within large stromal mucin; however, the number of signet ring cells in CC is significantly lower than in SRCC [5].

### Medullary carcinoma

MC is characterized by absent gland formation, syncytial growth, and a pushing border, often accompanied by abundant tumor-infiltrating lymphocytes [2]. It represents 0.006% of PDACs and is diagnosed at a median age of 65, with 66.7% of cases occurring in females [3]. Among PDAC subtypes, MC has the highest survival rates, with a median survival of 41 months and a 5-year survival rate of 33.3%, markedly better than cPDAC [3]. Microsatellite instability (MSI), frequently observed in MC, is associated with an elevated carcinogenesis risk [5, 27]. MSI is a genetic phenomenon resulting from impaired DNA mismatch repair, which leads to the accumulation of mutations in microsatellites, also known as short tandem repeats [28]. MSI is commonly associated with certain types of cancer, such as colorectal cancer (particularly in Lynch syndrome), as well as some endometrial and gastric cancers. It is also linked to a higher likelihood of response to immunotherapy [28, 29]. A family history of cancer is more commonly noted in patients with MC, emphasizing its relevance in the diagnostic process [30].

Comprehensive imaging data for MC remain sparse. MC is slightly more frequently found in the pancreatic head than in the body or tail [3]. Case reports describe MC as a well-defined tumor with central hypoattenuation on CT, hypointensity on T1WI, and peripheral enhancement [5, 31, 32]. The tumor’s center appears hyperintense, with a hypointense periphery on T2WI [5, 31]. No studies have reported findings on DCE studies, DWI, ADC, ^18^F-FDG-PET, or PET /CT for MC. Differentiation from cPDAC, particularly its poorly differentiated forms, remains challenging based on imaging alone [4].

### Adenosquamous carcinoma

ASqC is defined by at least 30% squamous differentiation within the tumor, with the remaining mass displaying varying degrees of glandular adenocarcinoma [2, 33]. It constitutes 0.8% of PDACs and is diagnosed at a median age of 68, with 45.2% of cases involving females [3]. The prognosis for ASqC is poorer than cPDAC, with a median survival of 7 months and a 5-year survival rate of 6.8% [3]. Although ASqC patients undergo resection more frequently (36.8%) than cPDAC patients (19.8%), their 5-year post-resection survival rate (15.7%) remains lower than that of resected cPDAC cases (19.1%) [3]

ASqC exhibits aggressive initial behavior, frequently presenting with liver metastases and lymphadenopathy [34, 35]. Surgical resection significantly improves median survival, and adjuvant chemotherapy or radiation therapy has been shown to prolong survival further [34, 36, 37]. In some cases, hypercalcemia of malignancy, potentially due to parathyroid hormone-related protein production by the squamous component, has been reported [38–40].

**Fig. 4:**
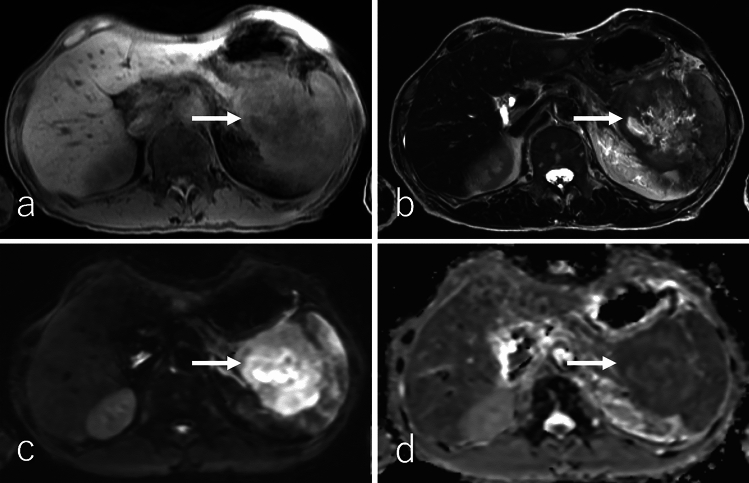
A 67-year-old male patient with adenosquamous carcinoma. **a** Fat-suppressed T1-weighted imaging (FS-T1WI), **b** T2-weighted imaging (T2WI), **c** diffusion-weighted imaging (DWI) with b value of 800 s/mm^2^, **d** apparent diffusion coefficient (ADC) map. The tumor is located in the pancreatic tail (arrow), measuring 8.0 cm in diameter, and demonstrates an expansile growth with well-defined margins. The tumor shows heterogeneous hypointensity on FS-T1WI and heterogeneous iso-to-hyperintensity on T2WI and DWI, and lower ADC values (1.09 × 10^−3^ mm^2^/s) relative to the pancreatic parenchyma

**Fig. 5:**
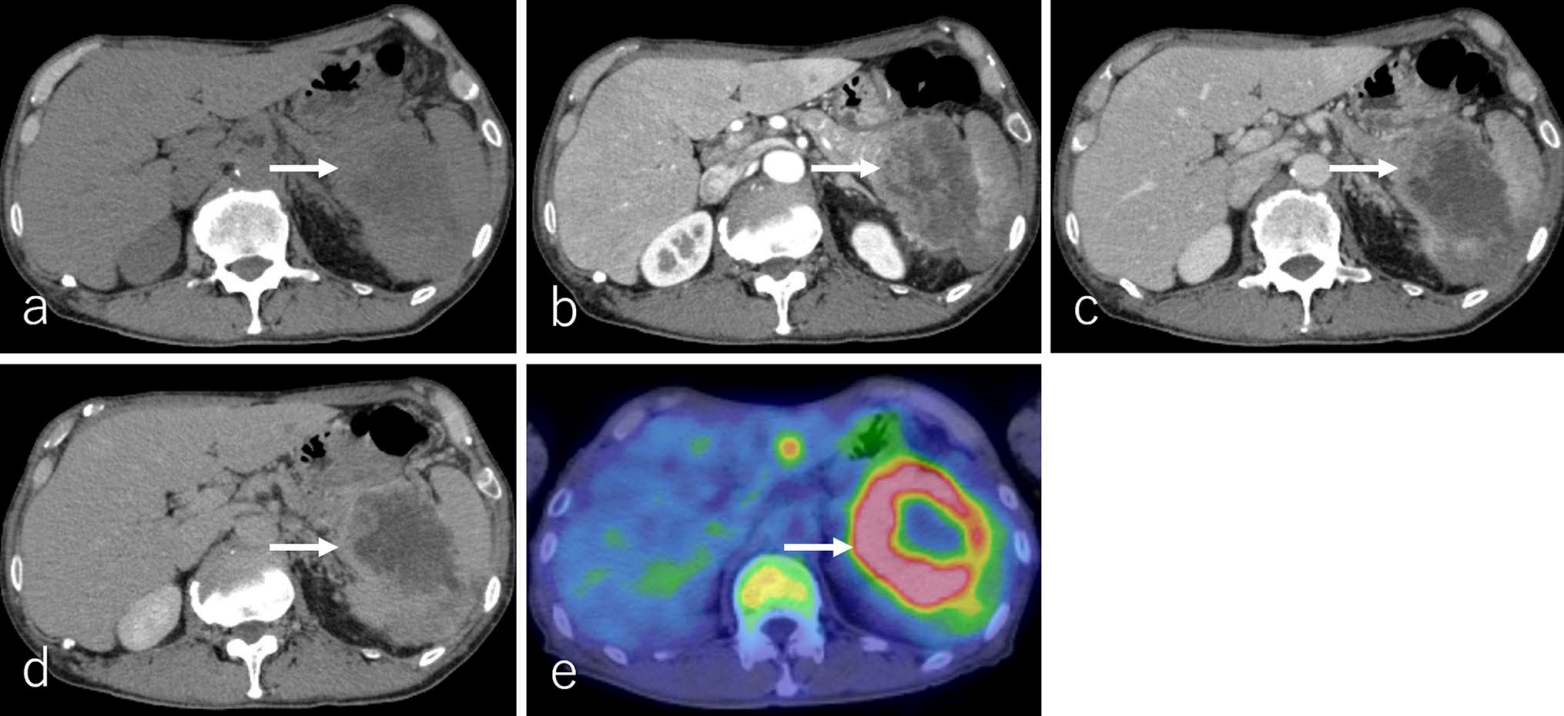
A 67-years-old male patient with adenosquamous carcinoma, the same patient as in Fig. 4. **a** Non-contrast-enhanced computed tomography (non-CECT) and dynamic contrast-enhanced CT in the **b** pancreatic phase, **c** portal phase, and **d** delayed phase, and **e** ^18^F-fluoro-2-deoxy-D-glucose positron emission tomography with CT (^18^F-FDG-PET/CT). The tumor is located in the pancreatic tail (arrow), measuring 8.0 cm in diameter. On non-CECT, the tumor exhibits a well-defined heterogeneous hypo – to isoattenuation. The central necrosis displays heterogeneous hypoattenuation without enhancement, consistent with central necrosis. In contrast, the solid peripheral region shows hypo- to isoattenuation on non-CECT and persistent enhancement on the delayed phase, often described as 'ring-like enhancement,' which is suggestive of a fibrous capsule and may serve as a useful predictive factor for ASqC. On ^18^F-FDG-PET/CT, the solid peripheral tumor shows increased ^18^F-FDG uptake, with a maximum standardized uptake value of 13.40 in the peripheral solid region of the tumor

Imaging of ASqC typically reveals a large, round, or lobulated tumor with either well- or ill-defined margins, often displaying extensive central necrosis—a distinguishing feature [5, 41–43]. ASqC occurs slightly more frequently in the pancreatic head than in the body or tail [3]. Central necrosis appears heterogeneously hypointense on T1WI, hyperintense on T2WI and DWI, with slightly higher ADC (Fig. 4a–d) and heterogeneous hypoattenuation on non-CECT (Fig. 5a) relative to the pancreatic parenchyma. Peripheral solid regions exhibit heterogeneous hypointensity on T1WI, iso- to hyperintensity on T2WI and DWI, and a lower ADC (Fig. 4a–d), with heterogeneous hypo- to isoattenuation on non-CECT (Fig. 5a) relative to the pancreatic parenchyma.

DCE studies show no enhancement in necrotic areas, while solid peripheral regions often demonstrate progressive "ring-like enhancement" suggestive of a fibrous capsule and useful for diagnosis (Fig. 5a–d) [43]. On ^18^F-FDG-PET or PET/CT, ASqC demonstrates increased ^18^F-FDG uptake in the peripheral solid regions and metastatic sites (Fig. 5e), aiding in differential diagnosis and assessment of adjacent tissue invasion [44]. Tumor thrombi, often associated with ductal dilation, are observed when ASqC invades nearby blood vessels or the pancreatic duct, correlating with a poorer prognosis [41–43]. Metastatic extrapancreatic squamous cell carcinoma should also be considered a potential mimic in the differential diagnosis ASqC [42, 43].

### Hepatoid carcinoma

HC is a rare pancreatic carcinoma defined by at least 50% of the tumor exhibiting histological and immunohistochemical evidence of hepatocellular differentiation [2, 45]. It accounts for 0.01% of PDACs and is diagnosed at a median age of 65, with 26.1% of cases involving females [3]. The median survival for HC is 5 months, with a 5-year survival rate of 26.1% [3]. Notably, HC demonstrates a better 5-year survival rate than cPDAC following surgical resection, though the prognosis for unresectable HC aligns with that of unresectable cPDAC [3]. HC is regarded as an aggressive tumor, frequently exhibiting early hematogenous metastasis to the liver and regional lymph-node involvement [5, 46]. Surgical resection is the primary treatment and significantly improves the likelihood of long-term survival, though the efficacy of additional postoperative therapies remains unclear [45].

Morphologically and immunohistochemically, HC closely resembles hepatocellular carcinoma (HCC) [45]. HC frequently produces alpha-fetoprotein (AFP), a biomarker for monitoring therapeutic response and detecting recurrence [45–47]. Additionally, des-gamma-carboxyprothrombin, also known as a protein induced by vitamin K absence or antagonist II, is elevated in some cases and may aid in early diagnosis and indicate a better prognosis [46].

Comprehensive imaging data for HC remain limited. Case reports describe HC are typically presenting with a well-delineated margin and a large, exophytic growth pattern [5, 46, 47]. Compared to cPDAC, HC is more commonly located in the pancreatic body or tail, although diffuse or multifocal lesions have also been reported [3, 5, 46]. HC demonstrates heterogeneous iso- to hyperintensity on T2WI and DWI [5, 47, 48]. No studies have yet reported about ADC findings for HC. Intratumoral microscopic fat content may occasionally be detected on chemical shift imaging [45]. DCE studies reveal hypoattenuation on non-CECT and hyperenhancement during the pancreatic phase, followed by washout in the portal or delayed phase. Capsular-like enhancement, a hallmark feature, is frequently observed and mirrors findings seen in HCC [45, 47]. On ^18^F-FDG-PET or PET/CT, HC shows intense ^18^F-FDG uptake in primary tumors and metastatic sites, though this finding is nonspecific [49].

HC and cPDAC exhibit distinct vascular imaging features, often aiding their differentiation. However, distinguishing HC from metastatic HCC can be challenging based on imaging alone. While metastatic HCC metastasis to the pancreas is rare and typically occurs during advanced stages of treatment, it should always be considered in the differential diagnosis [5]. Serum AFP levels, while useful for HC diagnosis, are nonspecific and can be elevated levels in other pancreatic tumors, such as PDACs, ACC, and PB [50]. Other hypervascular pancreatic tumors, including NEN and hypervascular ACC, should also be considered in the differential diagnosis due to their hyperenhancement patterns in DCE studies.

### Large cell carcinoma with rhabdoid phenotype

LCC-RP is an extremely rare and aggressive subtype of PDAC, characterized by pleomorphic neoplastic giant cells with abundant eosinophilic cytoplasm and rhabdoid inclusions [51]. Also referred to as undifferentiated rhabdoid carcinoma, LCC-RP is primarily described in case reports, with much of the existing literature focusing on its occurrence as a rare subtype of lung carcinoma [26, 52, 53]. It accounts for 0.003% of PDACs and is diagnosed at a median age of 61, with 60% of cases involving females [3]. This subtype has the poorest prognosis among all PDACs, with a median survival of 2 months and a 5-year survival rate of 0% [3].

LCC-RP is slightly more commonly found in the pancreatic body and tail than in the head [3].To date, no cases have reported specific radiological characteristics of LCC-RP, nor have studies described its differentiation from cPDAC.

### Undifferentiated carcinoma

UC is characterized by the absence of definitive differentiation, including glandular formation, mucin production, or keratinization [2, 54]. UC encompasses diverse growth patterns and coexisting cell types, such as spindle, giant, pleomorphic, and round cells [55]. According to the WHO classification, UC is categorized into three subtypes: anaplastic undifferentiated carcinoma, sarcomatoid undifferentiated carcinoma, and carcinosarcoma [2]. It accounts for approximately 0.1% of PDACs, with a median age of diagnosis at 67 years, and 41.3% of cases involve female patients [3].

The prognosis for UC is poor, with a median survival of 5 months and a 5-year survival rate of 11.8%, comparable to that of cPDAC [3]. However, survival varies among UC subtypes, with outcomes significantly improving following R0 or R1 resection compared to palliative surgery [56]. Serum tumor markers, including carbohydrate antigen 19–9 and CEA, are elevated in fewer than 20% of patients, reflecting the poorly differentiated nature of this malignancy [56].

Comprehensive reports on the imaging findings of UC are limited, but case studies describe nonspecific findings [5, 56–59]. UC typically exhibits extensive growth, frequently accompanied by areas of necrosis or degeneration. In over 50% of cases, cystic changes are observed, often visualized more effectively using endoscopic ultrasonography than other imaging modalities [59]. A study of 81 patients with UC revealed that 61% of tumors were located in the pancreatic head, with a median tumor diameter of 4 cm [56].

On imaging, UC is characterized by heterogeneous hypo- to isointensity on T1WI and heterogeneous hyperintensity on T2WI [57, 58]. No studies have yet detailed DWI or ADC findings for UC. DCE studies describe UC as having heterogeneous peripheral enhancement, most pronounced during the portal venous phase. This enhancement pattern likely reflects underlying necrosis or degeneration [5, 58, 60].

On ^18^F-FDG-PET or PET/CT, UC exhibits increased ^18^F-FDG uptake in the solid component; however, this finding is nonspecific [57, 58, 60]. Large tumor size and direct invasion into adjacent tissues are hallmarks of UC’s aggressive behavior and are critical features distinguishing it from cPDAC [5].

### Undifferentiated carcinoma with osteoclast-like giant cells

UCOGC comprises three distinct cell components: non-neoplastic osteoclast-like multinucleated giant cells, mononuclear histiocytic, and neoplastic mononuclear cell components [2]. It accounts for 0.1% of PDACs and is diagnosed at a median age of 66 years, with 50.6% of cases involving female patients [3]. The median survival for UCOGC is 12 months, with a 5-year survival rate of 28.4%, reflecting a better prognosis than cPDAC [3]. Long-term survival beyond 10 years has been documented in some cases [61]. Lymph-node metastasis and perineural invasion occur less frequently in UCOGC than in cPDAC [61]. Gross pathological examination typically reveals a solid or partially cystic tumor with a heterogeneous reddish-brown appearance attributed to hemorrhage and focal necrosis [5]. Notably, approximately 20% of UCOGC cases are associated with IPMN or MCN [5].

**Fig. 6:**
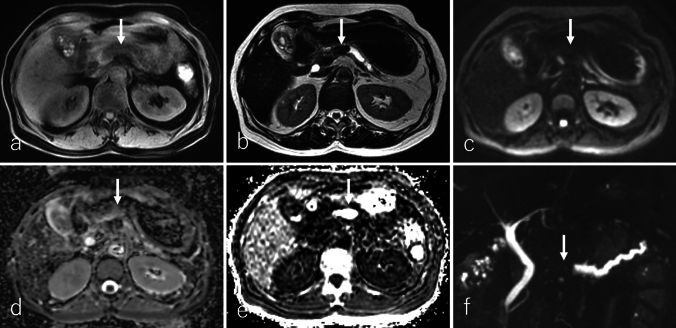
A 61-year-old female patient with undifferentiated carcinoma with osteoclast-like giant cells. **a** Fat-suppressed T1-weighted imaging (FS-T1WI), **b** T2-weighted imaging (T2WI), **c** diffusion-weighted imaging (DWI) with b value of 800 s/mm^2^, **d** apparent diffusion coefficient (ADC) map, **e** R2* map, and **f** magnetic resonance cholangiopancreatography (MRCP). The tumor is located in the pancreatic body (arrow), measuring 1.7 cm in diameter, and demonstrates an expansile growth with well-defined borders. The tumor shows heterogeneous hypointensity on FS-T1WI, T2WI, and DWI, and lower ADC values (0.86 × 10^−3^ mm^2^/s) relative to the pancreas parenchyma. *R*2* value is 300 per second which clearly revealed intratumoral hemorrhage. These findings are characteristic of the tumor and allowed for a preoperative differential diagnosis. MRCP shows that the tumor-induced obstruction of the pancreatic ducts results in upstream ductal dilation. These images are reproduced with permission from Sato et al., 'Undifferentiated carcinoma of the pancreas with osteoclast-like giant cells showing intraductal growth and intratumoral hemorrhage: MRI features'. Radiol Case Rep. 2019 Aug 14;14(10):1283–1287 © 2019 by Elsevier

UCOGC demonstrates distinctive imaging features. It typically presents as a well-defined solid tumor with smooth margins, expansive growth, and areas of necrosis or degeneration [5, 62, 63]. These tumors are generally larger, with 80% measuring over 5 cm, and they are more frequently located in the pancreatic head than in the body or tail [3, 5]. UCOGC exhibits hypointensity on T2WI (Fig. 6b), T2*-weighted imaging (T2*WI), and DWI (Fig. 6c), along with an elevated *R*2* value (Fig. 6e), indicative of hemosiderin deposition, which serves as a characteristic imaging feature [63–65].

**Fig. 7:**
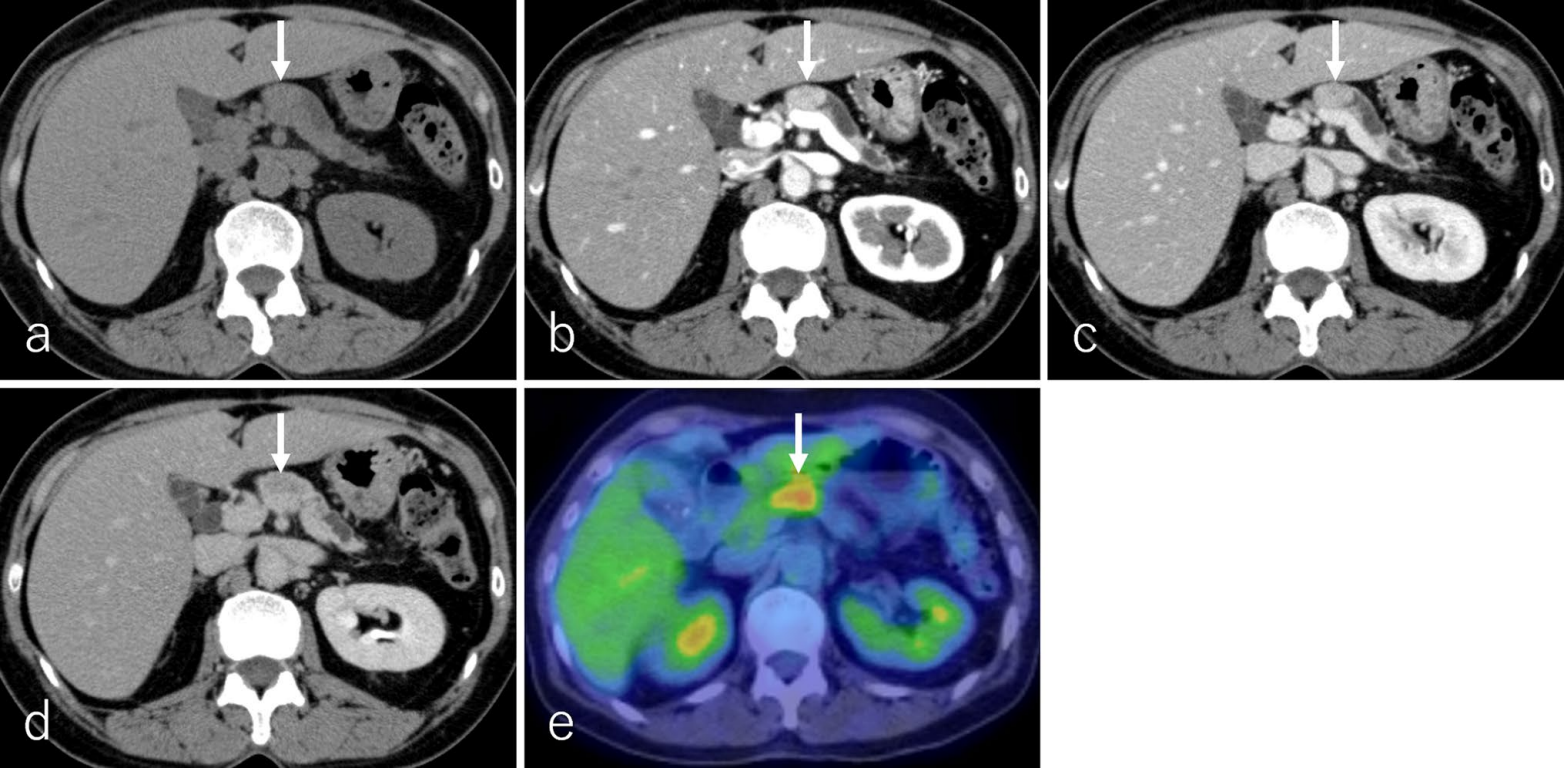
A 61-year-old female patient with undifferentiated carcinoma with osteoclast-like giant cells (UCOGC), the same patient as in Fig. 6. **a** Non-contrast-enhanced computed tomography (non-CECT) and dynamic contrast-enhanced CT in the **b** pancreatic phase, **c** portal phase, and **d** delayed phase, and **e** ^18^F-fluoro-2-deoxy-D-glucose positron emission tomography with CT (^18^F-FDG-PET/CT). The tumor is located in the pancreatic body (arrow), measuring 1.7 cm in diameter. The tumor demonstrates an expansile growth with well-defined margins. The tumor shows isoattenuation on non-CECT, while DCE-CT demonstrates that the tumor shows a homogenous enhancement on pancreatic and portal phases but slight hypoattenuation on the delayed phase. This enhancement pattern is not typical for large-sized UCOGCs. On ^18^F-FDG-PET/CT, the tumor shows increased ^18^F-FDG uptake, with a maximum standardized uptake value of 4.03. These images are reproduced with permission from Sato et al., 'Undifferentiated carcinoma of the pancreas with osteoclast-like giant cells showing intraductal growth and intratumoral hemorrhage: MRI features'. Radiol Case Rep. 2019 Aug 14;14(10):1283–1287 © 2019 by Elsevier

In DCE studies, UCOGC demonstrates weak enhancement relative to the pancreatic parenchyma across all phases, with prolonged enhancement noted in most cases [61, 63, 66]. However, two case reports describe small UCOGCs that exhibited homogeneous enhancement during the pancreatic and portal phases, followed by washout in the delayed phases (Fig. 7a–d) [62, 64]. Heterogeneous enhancement may correspond to areas of hemorrhage, necrosis, or degeneration. On ^18^F-FDG-PET or PET/CT, UCOGC demonstrates increased ^18^F-FDG uptake in the solid components, but this finding is nonspecific (Fig. 7e) [64, 67]. Like cPDAC, UCOGC often interrupts the main pancreatic duct, resulting in upstream ductal dilatation [64, 68].

Key imaging features distinguishing UCOGC from other PDAC subtypes include hypointensity on T2WI, T2*WI, and DWI and an elevated R2* value, which is atypical for cPDAC. Additionally, UCOGC often presents as a well-defined solid tumor with smooth margins and may display a hypointense rim on T2WI and T2*WI, indicative of a fibrous capsule. Intraductal or intravenous tumor growth is another important characteristic that can aid in differentiating UCOGC from cPDAC [5, 63, 64, 69].

## Other malignant pancreatic epithelial tumors

### Acinar cell carcinoma

ACC is a malignant epithelial neoplasm of the pancreas characterized by acinar cell differentiation [2]. It accounts for 1–2% of pancreatic tumors in adults and around 15% in pediatric patients [70]. According to the WHO classification, ACC includes three subtypes: mixed acinar-neuroendocrine carcinoma, mixed acinar-endocrine-ductal carcinoma, and mixed acinar-ductal carcinoma [2]. The median age of diagnosis for adult patients with ACC is 60, with 32.3% of cases occurring in females [70, 71]. The median survival for ACC is 47 months, with reported 5-year survival rates ranging from 36.2% to 72%. However, its prognosis remains debated, with some studies indicating better or worse outcomes than cPDAC [70, 72–74].

Approximately 16% of patients with ACC exhibit disseminated fat necrosis, often accompanied by polyarthralgia, which results from hypersecretion of lipase and trypsin [71, 75–77]. Pancreatic panniculitis (PP), a rare form associated with subcutaneous fat necrosis, is frequently linked to ACC [78]. PP typically manifests as painful, erythematous to violaceous nodules that may ulcerate and discharge oily, brown, viscous material due to liquefaction necrosis of adipocytes. These lesions primarily involve the lower extremities but may extend to the buttocks, trunk, arms, and scalp. Fat necrosis may also affect periarticular, abdominal, and intramedullary adipose tissue. Notably, in 40% of PP cases, skin symptoms precede abdominal manifestations associated with ACC or other pancreatic disorders by 1–7 months, including acute and chronic pancreatitis [78].

**Fig. 8:**
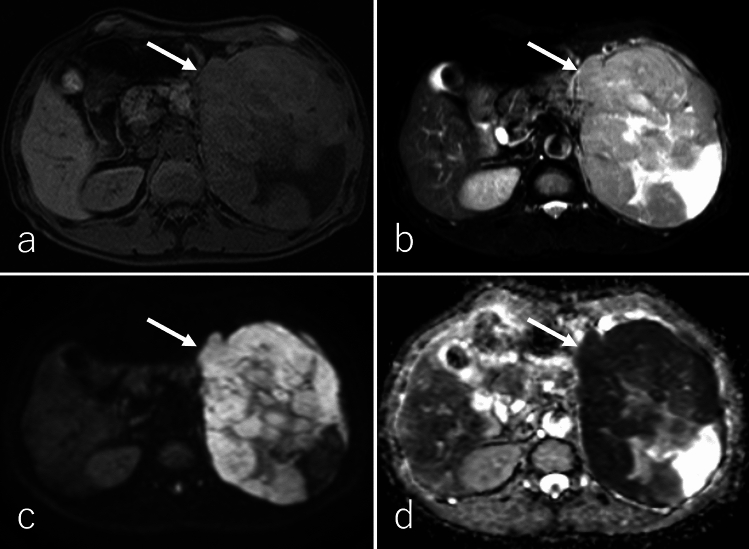
A 59-year-old male patient was diagnosed with acinar cell carcinoma (ACC). **a** Fat-suppressed T1-weighted imaging (FS-T1WI), **b** fat-suppressed T2-weighted imaging (FS-T2WI), **c** diffusion-weighted imaging (DWI) with *b*-value of 800 s/mm^2^, and **d** an apparent diffusion coefficient (ADC) map. The tumor is located in the pancreatic tail (arrow), measuring 15.0 cm in diameter, and exhibits expansile growth with well-defined margins. The tumor demonstrates heterogeneous hypointensity on FS-T1WI and heterogeneous iso- to hyperintensity on FS-T2WI and DWI. The solid region of the tumor shows lower ADC values (0.64 × 10⁻^3^ mm^2^/s), while the necrotic region exhibits higher ADC values (3.29 × 10⁻^3^ mm^2^/s) compared to the pancreatic parenchyma

**Fig. 9:**
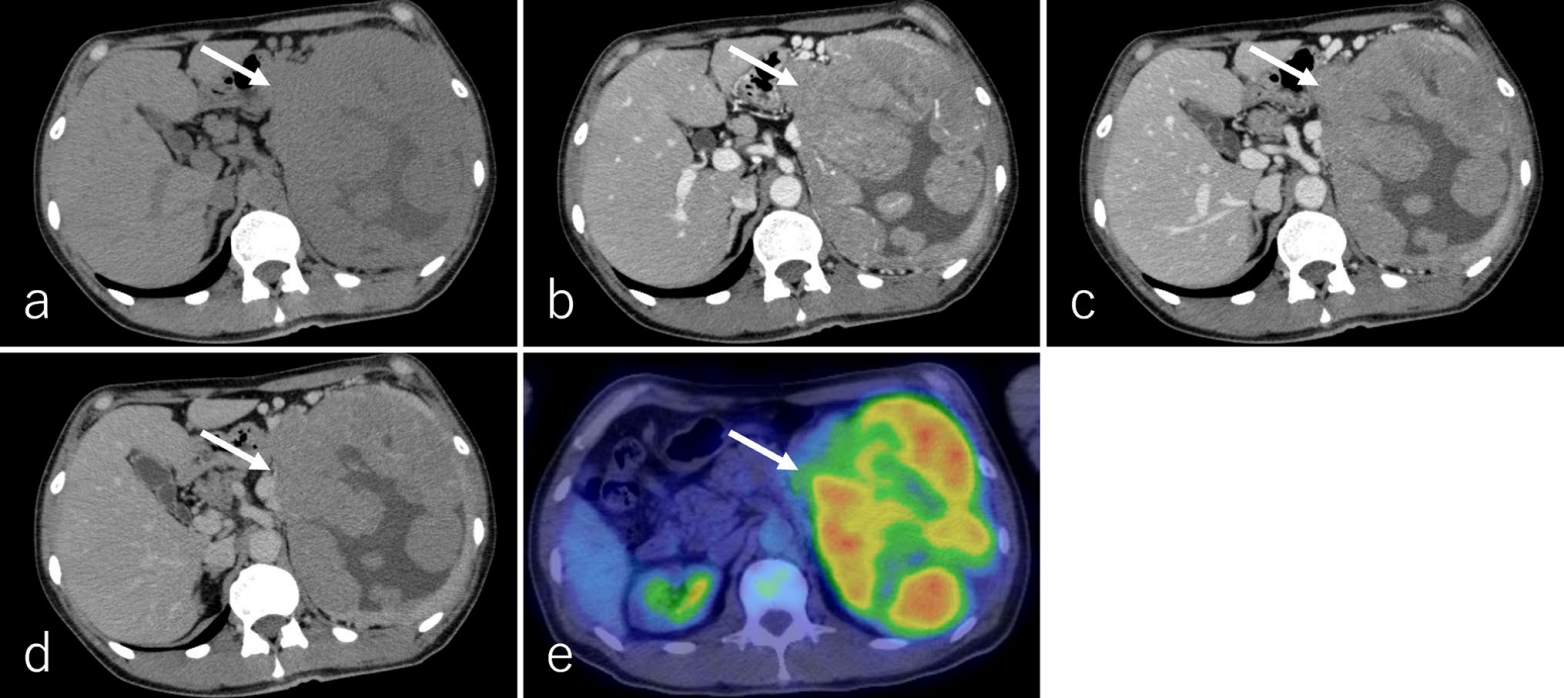
A 59-year-old male patient with acinar cell carcinoma, the same patient as in Fig. 8. **a** Non-contrast-enhanced computed tomography (non-CECT), **b** dynamic contrast-enhanced CT in the pancreatic phase, **c** portal phase, **d** delayed phase, and **e** ^1^⁸F-fluoro-2-deoxy-D-glucose positron emission tomography with CT (^1^⁸F-FDG-PET/CT). The tumor is located in the pancreatic tail (arrow), measuring 15.0 cm in diameter, and exhibits expansile growth with well-defined margins. The solid region of the tumor shows heterogeneous isoattenuation relative to the pancreatic parenchyma on non-CECT. In addition, it shows heterogeneous enhancement in the pancreatic phase with slight subsequent washout in the portal and delayed phases. Necrotic regions exhibit no contrast enhancement. On ^1^⁸F-FDG-PET/CT, the tumor shows increased ^18^F-FDG uptake, with a maximum standardized uptake value of 7.42 in the solid regions of the tumor

ACC typically presents as a well-defined, predominantly oval or round, exophytic, and large mass [79–82]. The tumor is generally dense and solid with minimal cystic changes, although larger lesions may demonstrate intratumoral necrosis or hemorrhage, occasionally accompanied by an enhancing capsule [79, 80, 82]. ACCs are most frequently located in the pancreatic head, followed by the tail and body [70, 83]. Calcifications are reported in 7% to 50% of cases [82]. On imaging, the solid component of ACC appears heterogeneous hypointense on T1WI, heterogeneously iso- to hyperintense on T2WI, hyperintense on DWI, and demonstrates a lower ADC relative to the pancreatic parenchyma (Fig. 8a–d) [80, 81, 84, 85]. On non-CECT, ACC exhibits heterogeneous isoattenuation compared to the pancreatic parenchyma (Fig. 9a) [69, 74].

ACCs exhibit variable enhancement patterns in DCE studies, depending on tumor size and necrosis or cystic changes (Fig. 9a–d). Most ACCs appear hypoattenuating or hypointense compared to the pancreatic parenchyma during both pancreatic and delayed phases, with slight and persistent enhancement. However, some cases may display hypervascularity [72, 79, 81, 85]. On ^18^F-FDG-PET or PET/CT, increased ^18^F-FDG uptake is typically observed in the solid component, excluding necrotic regions (Fig. 9e) [86]. Tumor thrombus formation may occur when ACC invades nearby blood vessels or the main pancreatic duct, often complicating management [79–81, 84].

**Fig. 10:**
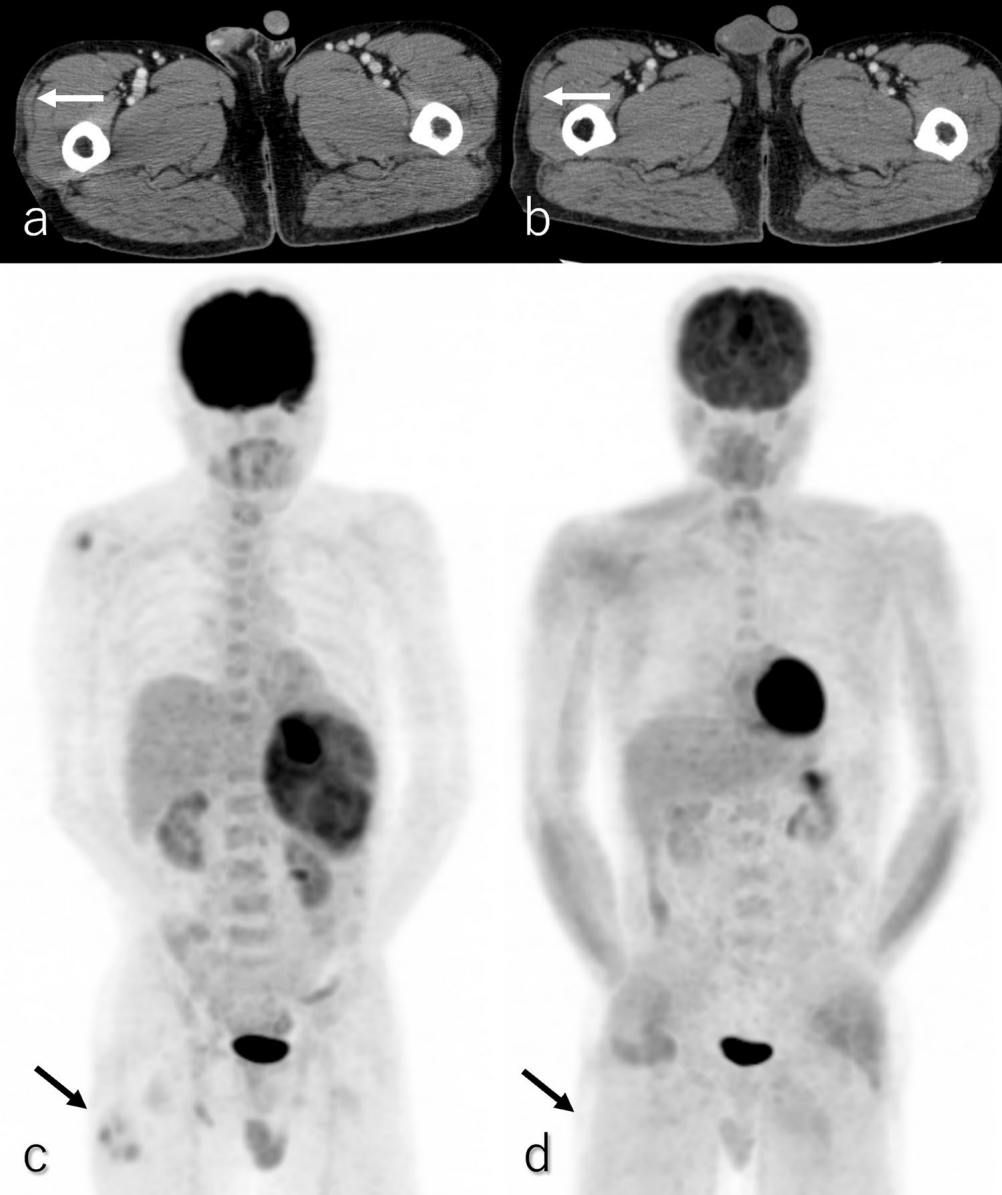
A 59-year-old male patient with acinar cell carcinoma (ACC), the same patient as in Figs. 8 and 9. **a** and **b** Dynamic contrast-enhanced CT (DCE-CT) in the pancreatic phase, **c** and **d** maximum intensity projection images of ^1^⁸F-fluoro-2-deoxy-D-glucose positron emission tomography with CT (^1^⁸F-FDG-PET/CT). Images (**b**) and (**d**) are obtained 7 months after the examinations shown in (**a**) and (**b**), respectively. The tumor is located in the pancreatic tail, measuring 15 cm in diameter, and exhibits expansile growth with well-defined margins. DCE-CT reveals an ill-defined, hazy appearance of the subcutaneous fat in the right thigh (arrow). ^1^⁸F-FDG-PET/CT demonstrates slightly increased ^1^⁸F-FDG uptake, with a maximum standardized uptake value of 2.14 in this region. These findings are consistent with pancreatic panniculitis (PP), characterized by subcutaneous fat necrosis associated with ACC. Following the surgical resection of the ACC, the ill-defined hazy appearance and ^1^⁸F-FDG uptake resolved, indicating an improvement in PP associated with the ACC

Key imaging findings for ACC include the degree of contrast enhancement, tumor boundary clarity, and tumor thrombus presence, which help distinguish it from cPDAC [70, 71]. However, certain overlapping features between ACC and cPDAC can complicate differentiation. Other differential diagnoses, such as NEN, PB, and SPN, present additional challenges, particularly since hypervascular ACC and NEN occasionally exhibit overlapping imaging features [70, 72]. Identification of PP offers significant diagnostic value for distinguishing ACC from cPDAC and other PDAC subtypes (Fig. 10a and c). Notably, symptoms improve clinically and radiologically following surgical resection of ACC as the tumor’s enzyme hypersecretion diminishes postoperatively (Fig. 10b and d).

### Pancreatoblastoma

PB is a malignant epithelial neoplasm of the pancreas characterized predominantly by acinar differentiation with squamoid nests [2]. Fewer than 200 cases have been reported in the literature, reflecting its rarity [2]. While the etiology of PB remains unclear, it is occasionally associated with genetic syndromes such as Beckwith–Wiedemann and familial adenomatous polyposis [2, 87, 88]. Genetic similarities between PB and hepatoblastoma have also been noted, suggesting shared developmental pathways [87].

PB is the most common pancreatic tumor in children, with most cases arising sporadically. It typically presents during the first decade of life, with a median age at diagnosis of 4 years and a slight male predominance [87, 88]. Pediatric patients with PB have an overall survival rate of approximately 80%, with complete surgical resection being a critical prognostic factor [87]. Conversely, patients with unresectable disease experience a survival period of less than 5 years [89, 90]. In adults, PB carries a poorer prognosis, with a median survival of 18.5 months, often due to metastasis and unresectable disease [90].

PB generally manifests as a well-defined, large, lobulated tumor with no preferential location within the pancreas [88, 91]. Calcifications are frequently observed and are described as rim-like or clustered [87, 88]. On imaging, PB typically demonstrates heterogeneous hypo- to isointensity on T1WI and heterogeneous hyperintensity on T2WI and DWI [92, 93]. The solid component exhibits a lower ADC relative to the pancreatic parenchyma [93]. In DCE studies, large PB tumors display heterogeneous enhancement, reflecting a combination of solid regions, cystic or necrotic areas, and septal enhancement. Smaller tumors, by contrast, demonstrate homogeneous enhancement with progressive intensification [88, 91, 93, 94]. On ^18^F-FDG-PET or PET/CT, adult cases of PB reveal increased ^18^F-FDG uptake; however, this finding is nonspecific [92, 94].

PB frequently encases major vascular structures, including the artery and portal vein, and invades adjacent organs, complicating therapeutic management [87]. At presentation, more than one-third of patients with PB exhibit metastases, with the liver, regional lymph nodes, and lungs being the most common sites of metastatic disease [87, 88].

Although imaging findings of PB are nonspecific, the patient’s age is a key factor in differential diagnosis. Other large intra-retroperitoneal lesions, such as neuroblastoma, lymphoma, and Wilms tumor, should be considered in pediatric patients. Elevated serum AFP, which correlates with tumor size, can aid in diagnosing PB [50, 87]. When differentiating PB from SPN in children, Yang et al. identified several distinguishing features, including age ≤ 5 years, elevated serum AFP levels, larger tumor size, ill-defined borders, calcifications, absence of hemorrhage, intratumoral vessels, peripancreatic vessel invasion, distant metastases, and lower ADC values [93].

### Solid-pseudopapillary neoplasm

SPN is a low-grade malignant pancreatic tumor characterized by poorly cohesive epithelial cells forming solid and pseudopapillary structures without specific differentiation along pancreatic epithelial lines [2]. It accounts for approximately 3% of all pancreatic tumors and 6% of exocrine pancreatic tumors [95–97]. This neoplasm predominantly affects females (90%), with a median age at diagnosis of 26 years, likely due to its progesterone dependency [96, 98]. While SPN generally carries a favorable prognosis, up to 15% of cases exhibit aggressive features, such as invasion of adjacent structures or metastatic disease [99]. Even in the presence of metastases, surgical resection of metastatic lesions often results in prolonged survival [99].

**Fig. 11:**
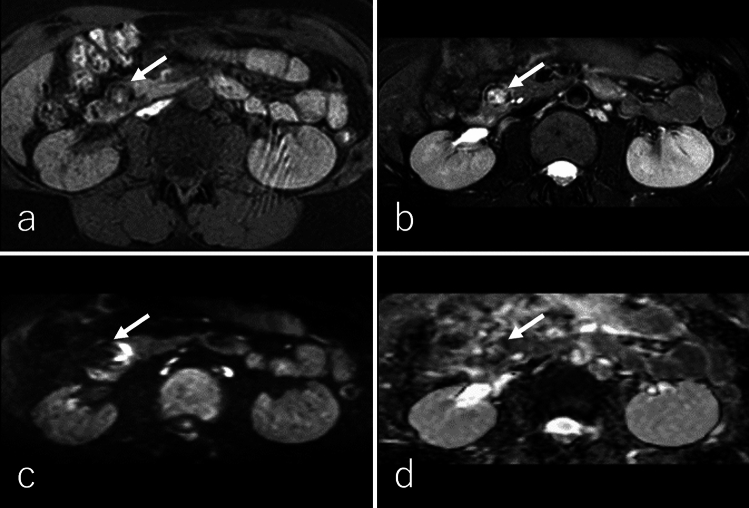
A 43-year-old male patient with a solid-pseudopapillary neoplasm. **a** Fat-suppressed T1-weighted imaging (FS-T1WI), **b** fat-suppressed T2-weighted imaging (FS-T2WI), **c** diffusion-weighted imaging (DWI) with *b* value of 800 s/mm^2^, and **d** an apparent diffusion coefficient (ADC) map. The tumor is located in the pancreatic head (arrow), measuring 2.0 cm in diameter, and exhibits expansile growth with well-defined margins. The central region of the tumor exhibits heterogeneous hyperintensity on FS-T1WI, FS-T2WI, and DWI, with an equivalent ADC values (1.46 × 10⁻^3^ mm^2^/s) relative to the pancreatic parenchyma. Additionally, the tumor demonstrates a hypointense rim on FS-T1WI, FS-T2WI, and DWI, corresponding to the peripheral rim calcification observed on CT (shown in Fig. 13)

**Fig. 12:**
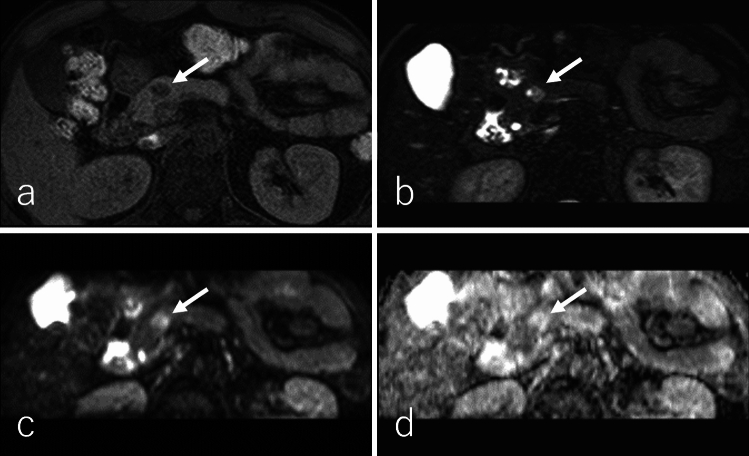
A 45-year-old male patient with solid-pseudopapillary neoplasm. **a** Fat-suppressed T1-weighted imaging (FS-T1WI), **b** fat-suppressed T2-weighted imaging (FS-T2WI), **c** diffusion-weighted imaging (DWI) with b value of 800 s/mm^2^, and **d** apparent diffusion coefficient (ADC) map. The tumor is located in the pancreatic head (arrow), measuring 1.5 cm in diameter, and exhibits expansile growth with well-defined margins. The tumor exhibits heterogeneous hypointensity on FS-T1WI, heterogeneous hyperintensity on FS-T2WI, and DWI, with higher ADC values (3.10 × 10⁻^3^ mm^2^/s), relative to the pancreatic parenchyma. Additionally, the tumor demonstrates a small cystic component on the right side of the tumor on FS-T2WI

**Fig. 13:**
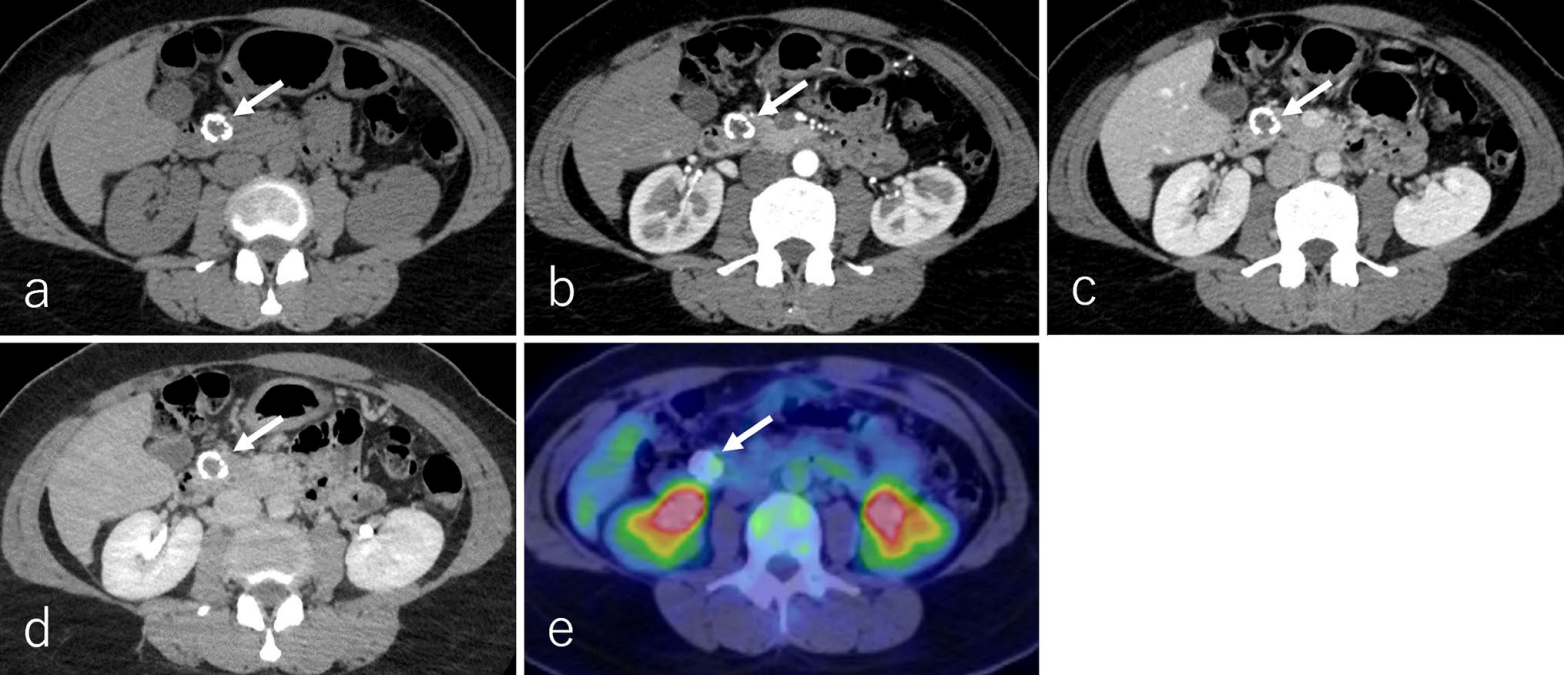
A 43-year-old female patient was diagnosed with a solid-pseudopapillary neoplasm, the same patient as in Fig. 11. **a** Non-contrast-enhanced computed tomography (CT), **b** dynamic contrast-enhanced CT in the pancreatic phase, **c** portal venous phase, **d** delayed phase, and **e** ^1^⁸F-fluoro-2-deoxy-D-glucose positron emission tomography with CT (^1^⁸F-FDG-PET/CT). The tumor is located in the pancreatic head (arrow), measuring 2.0 cm in diameter, and exhibits expansile growth with well-defined margins with peripheral rim calcification. The central region of the tumor demonstrates poor enhancement during the pancreatic phase, with slightly persistent enhancement observed in the portal venous and delayed phases. On ^1^⁸F-FDG-PET/CT, the tumor shows mildly increased ^18^F-FDG uptake, with a maximum standardized uptake value of 2.51

SPN typically presents as a well-defined, large tumor at diagnosis, with a mean diameter of 9–10 cm, often containing necrotic, solid, and hemorrhagic regions [95, 100–103]. However, with increased utilization of cross-sectional imaging, smaller tumors (< 5 cm) are now being detected more frequently [104]. The pancreatic head is the most common site of SPN, followed by the tail [102]. Imaging findings in a study of 41 patients revealed that SPNs exhibit heterogeneous hypointensity (41%) or homogeneous hypointensity (47%) on T1WI, with 12% demonstrating heterogeneous hyperintensity (Figs. 11a and 12a) [103]. On T2WI, 94% of SPNs demonstrated heterogeneous hyperintensity (Figs. 11b and 12b), while 6% displayed homogeneous hyperintensity [103]. Hemorrhagic regions occasionally produce a fluid–fluid level on T2WI and DWI [93, 95]. The solid regions of SPNs demonstrate heterogeneous hyperintensity on DWI (Figs. 11c and 12c) and an equal or higher ADC (Figs. 11d and 12d) relative to the pancreatic parenchyma [93, 101]. Peripheral calcification is present in approximately one-third of SPNs, and hemorrhagic regions are observed in 50% of cases (Fig. 13) [100, 101].

**Fig. 14:**
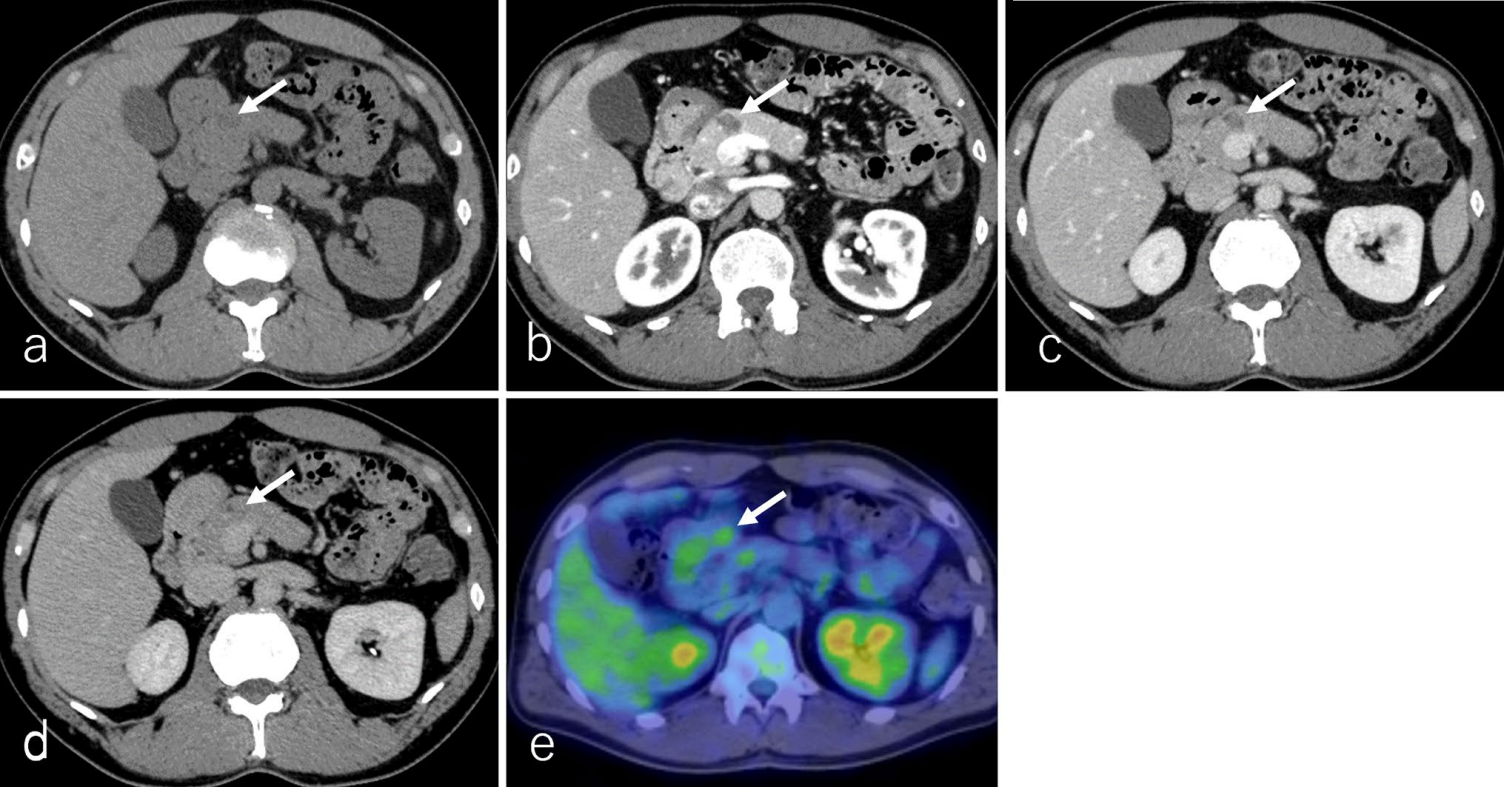
A 45-year-old female patient with solid-pseudopapillary neoplasm, the same patient as in Fig. 10. **a** Non-contrast-enhanced computed tomography (CT), **b** dynamic contrast-enhanced CT in the pancreatic phase, **c** portal phase, **d** delayed phase, and **e** ^1^⁸F-fluoro-2-deoxy-D-glucose positron emission tomography with CT (^1^⁸F-FDG-PET/CT). The tumor is located in the pancreatic head (arrow), measuring 1.5 cm in diameter, and exhibits expansile growth with well-defined margins. The tumor shows poor enhancement in the pancreatic phase with persistent enhancement in the portal and delayed phases. Tumor necrosis and calcification are not evident. On ^1^⁸F-FDG-PET/CT, the tumor shows mildly increased ^18^F-FDG uptake, with a maximum standardized uptake value of 2.44

DCE studies demonstrate that SPNs lack enhancement in areas dominated by hemorrhage and calcification (Fig. 13). In contrast, the solid regions exhibit heterogeneous peripheral enhancement or, less commonly, homogeneous enhancement during the pancreatic phase. Progressive but incomplete enhancement is observed during the portal venous and delayed phases (Fig. 14) [95, 102]. In larger tumors, the mass effect may cause upstream dilatation of the main pancreatic duct or its side branches, although secondary pancreatic ductal dilation is uncommon [103]. On ^1^⁸F-FDG-PET or PET/CT, SPNs with high cellularity exhibit mild-to-intense ^18^F-FDG uptake, whereas tumors with low cellularity demonstrate minimal ^1^⁸F-FDG uptake (Figs. 13e and 14e) [105].

The diagnosis of SPN is often straightforward when the patient's age, gender, and characteristic imaging findings are considered. However, atypical SPN cases pose diagnostic challenges, particularly in solid SPNs without degeneration or hemorrhage, which may mimic ACC, NEN, or metastatic tumors. Cystic SPNs should be differentiated from cystic pancreatic tumors such as CC and MCN. In pediatric patients, differentiation from PB is critical due to overlapping radiological features [93]. Additional diagnostic challenges arise in male patients or younger children (< 10 years) with SPNs. Morphological and functional imaging findings, when combined with clinical context, significantly aid in establishing an accurate diagnosis.

## Conclusion

Identifying characteristic radiological features specific to subtypes of PDAC and other MPETs can significantly enhance differential diagnosis. By adopting a systematic approach to imaging evaluation and correlating findings with clinical and histopathological data, radiologists can contribute to improved diagnostic precision and patient outcomes. Future studies should aim to further delineate unique imaging features of these rare tumors, supporting more reliable differentiation in clinical practice.
